# Mentoring strategies to support diversity in research-focused junior faculty: A scoping review

**DOI:** 10.1017/cts.2022.474

**Published:** 2022-10-06

**Authors:** Joni S. Williams, Rebekah J. Walker, Kaylin M. Burgess, L. Aubree Shay, Susanne Schmidt, Joel Tsevat, Jennifer A. Campbell, Aprill Z. Dawson, Mukoso N. Ozieh, Shane A. Phillips, Leonard E. Egede

**Affiliations:** 1 Department of Medicine, Division of General Internal Medicine, Medical College of Wisconsin, Milwaukee, WI, USA; 2 Center for Advancing Population Science, Medical College of Wisconsin, Milwaukee, WI, USA; 3 UTHealth School of Public Health in San Antonio, San Antonio, TX, USA; 4 Department of Population Health Sciences, Joe R. and Teresa Lozano Long School of Medicine, University of Texas Health Science Center at San Antonio, San Antonio, TX, USA; 5 ReACH Center and Department of Medicine, Joe R. and Teresa Lozano Long School of Medicine, University of Texas Health Science Center at San Antonio, San Antonio, TX, USA; 6 Department of Medicine, Division of Nephrology, Medical College of Wisconsin, Milwaukee, WI, USA; 7 Department of Medicine, Clement J. Zablocki VA Medical Center, Milwaukee, WI, USA; 8 Department of Physical Therapy, College of Applied Health Sciences, Center for Clinical and Translational Sciences, University of Illinois at Chicago, Chicago, IL, USA

**Keywords:** Mentoring, diversity, junior faculty, clinical research, scoping review

## Abstract

**Objective:**

The purpose of this scoping review is two-fold: to assess the literature that quantitatively measures outcomes of mentorship programs designed to support research-focused junior faculty and to identify mentoring strategies that promote diversity within academic medicine mentoring programs.

**Methods:**

Studies were identified by searching Medline using MESH terms for mentoring and academic medicine. Eligibility criteria included studies focused on junior faculty in research-focused positions, receiving mentorship, in an academic medical center in the USA, with outcomes collected to measure career success (career trajectory, career satisfaction, quality of life, research productivity, leadership positions). Data were abstracted using a standardized data collection form, and best practices were summarized.

**Results:**

Search terms resulted in 1,842 articles for title and abstract review, with 27 manuscripts meeting inclusion criteria. Two studies focused specifically on women, and four studies focused on junior faculty from racial/ethnic backgrounds underrepresented in medicine. From the initial search, few studies were designed to specifically increase diversity or capture outcomes relevant to promotion within academic medicine. Of those which did, most studies captured the impact on research productivity and career satisfaction. Traditional one-on-one mentorship, structured peer mentorship facilitated by a senior mentor, and peer mentorship in combination with one-on-one mentorship were found to be effective strategies to facilitate research productivity.

**Conclusion:**

Efforts are needed at the mentee, mentor, and institutional level to provide mentorship to diverse junior faculty on research competencies and career trajectory, create a sense of belonging, and connect junior faculty with institutional resources to support career success.

## Introduction

Effective mentorship is the cornerstone of faculty development in academic medicine and is shown to not only enhance career development but also strengthen institutional and department support for junior faculty [1–4]. While there is neither a universal definition of mentoring nor qualifications for who can serve as a mentor, when effective mentorship can significantly enhance professional identity, personal competence, research productivity, and faculty advancement [1,5–7]. Specific examples of career successes and professional development among junior faculty that have resulted from successful mentoring relationships include advancement in the promotion and tenure process, acquisition of independent grant funding, appointment to leadership positions, and increased productivity with regards to peer-reviewed publications [1,2,6]. In addition, personal development, career satisfaction, quality of life, and self-esteem have also been reported [1,2,6].

Faculty seeking to conduct research as a part of their academic medicine career face an additional layer of difficulty in defining and succeeding in their career path [8–10]. Training in research methods, dedicated time for research, and mentorship specific to their research role are noted as important facilitators of success [8–10]. In fact, the National Institute of Health and the Institute of Medicine identified research mentoring as critically important to increase the capacity of clinical and translational researchers in the USA [10,11].

However, diversity, equity, and inclusion in science and the scientific workforce are necessary for academic medicine to be at the forefront of innovation in the future [8,12,13]. Diverse scholars, including junior faculty who are women and junior faculty from racial and ethnic backgrounds underrepresented in medicine, need access to training, learning communities, and mentorship, in addition to the removal of structural inequities within institutions [4,12,14,15]. For example, effective mentoring among women can be complicated by organizational factors such as the lack of available mentors with mutual interests or with research experience and by personal and relationship dynamics such as differences in age, gender, culture, and past experiences [4]. In addition, the power differential posed by the traditional hierarchical structure of mentor–mentee relationships has served as a barrier to mentoring among women [4]. Similarly, junior faculty from racial and ethnic backgrounds underrepresented in medicine can experience challenges such as biases, discrimination, and prejudice, resulting in feelings of isolation [15]. They can also experience a lack of confidence and increased self-doubt, leading to higher attrition from academic careers at institutions of higher education [15]. Fortunately, mentoring can serve as the mechanism for providing support, removing inequities, and ultimately, facilitating career development and success for junior faculty [4,15]. Factors such as the availability, expertise, and responsiveness of the mentor; mutuality; protected time for mentor–mentee contact; and supportive relationships have been shown to facilitate personal and career development among junior faculty [2,4,16].

Given the demonstrated benefits of successful and effective mentor–mentee relationships, and the need to enhance mentorship for diverse junior faculty focused on incorporating research into their academic medical career, a review of successful mentoring strategies are warranted. While a number of reviews on mentorship in medicine exist, there are two primary gaps, that this review aims to fill. First, this review focuses on mentorship programs designed for research-focused junior faculty, defined as junior faculty conducting clinical or translational research as part of their academic medicine career. As such, mentorship regarding administrative time, clinical expertise, or educational efforts was not a focus of this review. Secondly, this review focuses on mentorship programs that quantitatively measured outcomes related to academic medical career trajectory, satisfaction within a research career, and research productivity, including grants and manuscripts. Finally, this review aimed to identify successful strategies that could be used to enhance existing and inform new mentorship programs that aim to increase diversity in academic medicine and research. As such, mentorship programs were not excluded for a lack of diversity, but it was noted if adaptations were made to specifically promote diversity. Therefore, the purpose of this review is two-fold: (1) to assess the literature that quantitatively measures outcomes of mentorship programs designed to support research-focused junior faculty in academic medical settings and (2) to identify mentoring strategies that promote diversity.

## Methods

### Search Term Selection, Eligibility Criteria, and Search Strategy

We followed PRISMA guidelines for conducting and reporting systematic reviews as a guide in conducting this scoping review [17]. A reproducible method was used to identify published papers on the measurable impact of mentoring on career success. Career success was defined to include career trajectory (including promotion through academic ranks), career satisfaction, quality of life, research productivity (grant funding, peer-reviewed publications), and leadership position(s) attainment in academic medicine.

Studies were identified by searching the Medline database using PubMed through August 3, 2022. The search terms were based on search strategies identified in published systematic reviews for mentoring [18,19] and careers in academic medicine [19–21] with final search terms simplified to include only MESH terms. Search terms and resulting number of articles identified with each step of the search can be found in Table 1.

**Table 1.** Structure of search and search terms for PubMed

Search #	Search Terms	Number of articles found
1	“Mentoring”[MESH] **OR** “Mentors”[MESH]	7,911
2	“Academic Medical Centers”[MESH] **OR** “Education, Medical”[MESH] **OR** “Faculty, Medical”[MESH] **OR** “Schools, Medical”[MESH] **OR** “Biomedical Research”[MESH]	316,513
3	#1 **AND** #2	1,842

Eligibility criteria were created based on the PICOS (participants, interventions, comparisons, outcomes, study design) approach recommended by PRISMA guidelines [17]. Inclusion criteria included studies addressing, (a) junior faculty, (b) in research-focused faculty positions, (c) receiving mentorship, (d) in an academic medical center, (e) in the USA, (f) with outcomes collected to measure career success (career trajectory, career satisfaction, quality of life, research productivity, leadership position attainment). Studies that were not published in English, used qualitative methods only, and systematic reviews were excluded. To account for articles that did not explicitly characterize strategies as being focused on mentorship efforts to increase diversity, this search included all mentee characteristics for a comprehensive assessment of available literature on mentorship programs.

### Study Selection and Data Collection

**Fig. 1.**
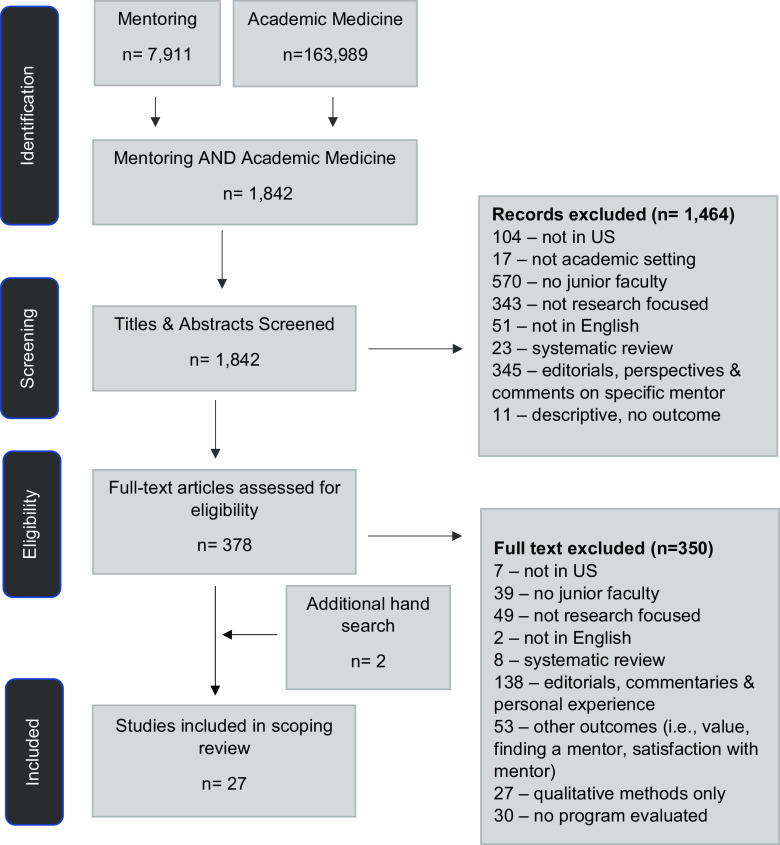
Flow diagram for eligible article selection.

Figure 1 outlines the strategy used to identify eligible articles. Titles and abstracts were initially screened by three reviewers (JSW, RJW, and KMB) to ensure articles were conducted in US academic settings, with junior faculty, and focused on research careers. Two reviewers (JSW & RJW) independently screened 50% of the articles and double screened a random sample of the second half to ensure reviewer agreement. One other reviewer (KMB) independently screened 27% of the articles to ensure all articles meeting the inclusion criteria were identified; these articles were double screened by one of the initial reviewers (JSW) to ensure reviewer agreement. Any differences were addressed by discussion with the larger author team. Examples of reasons for excluding articles labeled as “not academic setting” included studies conducted in community practice, industry, or comparisons between academic and community settings. Examples of reasons for excluding articles labeled as “not junior faculty” included studies collecting information on mentorship of residents, medical students, interns, undergraduates, senior faculty, or post-docs, or comparisons between junior faculty and students/interns/residents. Examples of reasons for excluding articles labeled as “not research focused” included mentorship on teaching, clinical skills, clinical supervision, procedures, residency directors, or continuing medical education.

Following title and abstract screening, full-text articles were further assessed to ensure measurable outcomes were collected and reported. Two independent reviewers (JSW and RJW) used the same process of random sample of double review to ensure reviewer agreement of the full-text articles. Examples of reasons for excluding articles labeled as “other outcomes” that did not measure career success included editorials, perspectives, articles describing experience with a specific mentor, commentaries, presidential address summaries, personal experience, description of mentoring without an evaluation, measurement of satisfaction with finding a mentor, or measurement of whether individuals had a mentor. For quantitative studies, papers that described the importance of mentorship or the state of mentorship at an institution but did not provide an evaluation of the effectiveness of that mentorship were excluded. An additional hand search (JSW) using the same inclusion criteria yielded two additional articles that were included in this review.

Data were then abstracted from each article by using a standardized data collection form that included the following headings: author, year, study goal/objective, study population, study setting, strategy/intervention/approach deployed, and main findings/best practices. Data collected from each eligible article are presented in Tables 1 and 2. Quality of the manuscripts was not assessed due to the wide variety of study designs and outcomes collected in each study. Similarly, no quantitative analysis of results was conducted due to the variety of outcome measurements.

**Table 2.** Summary of articles included in review of mentorship for research-focused junior faculty

Article	Study goal/objective	Study population	Study setting	Strategy/intervention/approach deployed
*Career trajectory*				
Blood *et al.* [2]	Examine the role of academic rank, research focus, parenting and part-time work on mentoring importance, needs, and gaps	1,179 women faculty	Harvard Medical School and Harvard School of Dental Medicine	Experience with mentoring and mentoring needs (i.e., developing and achieving career goals, clinical skills, research skills, writing and publishing articles, obtaining grant funding, etc.)
Boutjdir *et al.* [22]	Assess the 12-year outcomes of the National Heart, Lung and Blood Institute (NHLBI) Programs to Increase-Diversity-Among-Individuals-Engaged in Health-Related Research (PRIDE) focused on cardiovascular disease (CVD)	136 under-represented in medicine (URM) and/or individuals with disabilities who are junior faculty from 93 US institutions during 2006–2018	Two academic institutions [State University of New York Downstate-Medical-Center at Brooklyn (SUNY-Downstate) and Washington University School of Medicine (WUSM)]	Longitudinal cohort conducted over two consecutive summers and the intervening academic year. Included a mid-year meeting, and a workshop at NIH. Program focused on (1) didactic CVD courses; (2) mentoring, career development, academic-promotion, networking; and (3) grant proposal development, review, and funding strategies
Bredella *et al.* [23]	Implement and assess the impact of a mentoring program for early career faculty in an academic radiology department	126 clinical faculty	Academic radiology department at Massachusetts General Hospital and Harvard Medical School	Paired mentoring with a senior radiologist outside of the division, structured one-on-one mentoring, a mentoring network, and peer mentoring
Efstahiou *et al.* [24]	Assess the short and long-term impact of a formal mentoring program on junior faculty satisfaction and productivity	15 mentees and 23 controls	Departments of Radiation Oncology and Anesthesia, Critical Care, and Pain Management	Mentor pairs, three formal training sessions, informal meetings, formalized action plans
Hackworth *et al.* [25]	Examine the association between mentoring and academic productivity for junior faculty members in a pediatric academic health center	319 Instructors and Assistant Professors for calendar year 2017	Cincinnati Children’s Hospital Medical Center	Assignment of a primary mentor and a Career Development Committee (CDC)
Prendergast *et al.* [26]	Evaluate an enhanced peer mentoring program	22 faculty (4 senior peer advisors and 18 junior faculty)	Academic emergency medicine hospital in Chicago, IL	Facilitated peer group-mentoring platform designed to address self-efficacy, relatedness/inclusion, engagement, and institutional commitment to junior faculty. Senior peer advisors advised groups of 4–5 junior faculty formed around primary area of academic focus (education, research, clinical). Groups created action plans at first meeting and met quarterly. Mentees also gave innovations in scholarship presentations for faculty external to mentoring program.
*Career satisfaction*				
Chen *et al.* [27]	Develop, implement, and evaluate a multi-faceted pediatric mentoring program to promote retention and satisfaction	8 mentor–mentee dyads	Department of Pediatrics at Stanford University School of Medicine	Multi-component intervention that included (1) individualized mentor-mentee meetings outside the academic division, (2) quarterly didactic workshops on career-related topics, (3) peer mentoring group meetings, and (4) individualized assistance with grant writing and review
Nagarur *et al.* [28]	Evaluate structured mentorship program for junior hospitalists	16 mentees wit >0.5 FTE and 3 years or less hospitalist experience	Hospital Medicine Unit of Massachusetts General Hospital	Three training sessions over 9 months for mentors and mentees on maximizing mentorship success. Mentee–mentor pairs expected to meet minimum of two times and developing action plans with individualized goals
Travis *et al.* [29]	Investigate influence of mentoring on career satisfaction in gastroenterology and if it differed by gender	Faculty responding to online survey	Survey conducted by American College of Gastroenterology for all physician members	Factors associated with effective mentoring included frequent meetings, career mentoring, and mentors at rank of professor
Varkey *et al.* [30]	Evaluate a facilitated peer mentoring program	19 women faculty with rank of Instructor or Assistant Professor and 4 women faculty facilitators with rank of Associate Professor or Professor	Department of Medicine at Mayo Clinic in Rochester, Minnesota	Facilitated peer mentoring groups matched based on research and clinical interests, with orientation session followed by group meetings at least monthly focused on manuscript development over 12 months
Walensky *et al.* [31]	Assess mentorship experiences among faculty of large academic department of medicine	553 faculty responded to online survey	Faculty members in Massachusetts General Hospital Department of Medicine	Assessed having a mentor, role of mentor, and mentorship quality
*Quality of life*				
Blood *et al.* [2]	Examine the role of academic rank, research focus, parenting and part-time work on mentoring importance, needs, and gaps	1,179 women faculty	Harvard Medical School and Harvard School of Dental Medicine	Experience with mentoring and mentoring needs (i.e., developing and achieving career goals, clinical skills, research skills, writing and publishing articles, obtaining grant funding, etc.)
*Academic productivity (publications and grants)*				
Boutjdir *et al.* [22]	Assess the 12-year outcomes of the National Heart, Lung and Blood Institute (NHLBI) Programs to Increase-Diversity-Among-Individuals-Engaged in Health-Related Research (PRIDE) focused on cardiovascular disease (CVD)	136 under-represented in medicine (URM) and/or individuals with disabilities who are junior faculty from 93 US institutions during 2006–2018	Two academic institutions (State University of New York Downstate-Medical-Center at Brooklyn (SUNY-Downstate) and Washington University School of Medicine (WUSM)	Longitudinal cohort conducted over two consecutive summers and the intervening academic year. Included a mid-year meeting, and a workshop at NIH. Program focused on (1) didactic CVD courses; (2) mentoring, career development, academic promotion, networking; and (3) grant proposal development, review, and funding strategies
Bredella *et al.* [23]	Implement and assess the impact of a mentoring program for early career faculty in an academic radiology department	126 clinical faculty	Academic radiology department at Massachusetts General Hospital and Harvard Medical School	Paired mentoring with a senior radiologist outside of the division, structured one-on-one mentoring, a mentoring network, and peer mentoring
Brown *et al.* [32]	Increase and promote diversity among researchers and participants in HIV-related scholarship via the Mid-Atlantic Center for AIDS Research Consortium (MACC) Scholars Program	4 URM scholars	Johns Hopkins University, George Washington University, and the University of Pennsylvania	Each junior investigator was paired with a senior mentor and awarded a grant to develop a pilot study on an HIV-related topic (prevention, treatment, or care)
Brownson *et al.* [33]	Promote intervention strategies and application of dissemination and implementation science tools in the Mentored Training for Dissemination and Implementation Research in Cancer (MT-DIRC) Program	55 fellows	Washington University	2-year training institute comprised of (1) two annual summer institutes and conferences that incorporated didactic group and individual instruction, (2) individualized mentoring, and (3) pilot funding
Burns *et al.* [34]	Understand the effect of an intense mentoring program on junior investigators’ preparation for patient-oriented clinical research careers	140 early career physician-scientists from the first 7 years (2003–2010) of the Clinical Research Training Institute (CRTI)	Multiple institutions throughout the USA and Canada	Yearlong educational and mentoring experience including (1) weeklong workshop for direct interaction between faculty mentors and trainees for at least 13 h each day, (2) didactic component on conducting patient-oriented clinical research projects and processes (acquiring funding, addressing regulatory issues and ethical outcomes), and (3) life-work balance
Chou *et al.* [35]	Assess the impact of the Oklahoma IDeA Network of Biomedical Research Excellence (OK-INBRE) research support and mentoring program	63 new and early-stage investigators throughout OK in the OK-INBRE Research Project Investigator (RPI) Award Program	Research Intensive Institutions in Oklahoma (University of OK Health Sciences Center, University of OK, OK State University, OK Medical Research Foundation)	Formalized mentoring and grant funding ($100,000 direct costs per year) and application peer review by a panel of senior investigators funded by the NIH and/or serving on NIH review panels
Daley *et al.* [36]	Create a cohort of investigators engaged in health disparities research, scholarship, and practice and increase funding in health disparities research	19 Underrepresented minority (URM) junior faculty members at the assistant professor level	San Diego Center of Excellence in Partnership for Community Outreach, Research on Disparities in Health and Training (EXPORT Center)	Focus of program in two initiatives: (1) support of URM junior faculty career development and (2) funding for pilot research grants in health disparities
Efstahiou *et al.* [24]	Assess the short and long-term impact of a formal mentoring program on junior faculty satisfaction and productivity	15 mentees and 23 controls	Departments of Radiation Oncology and Anesthesia, Critical Care, and Pain Management	Mentor pairs, three formal training sessions, informal meetings, formalized action plans
Freel *et al.* [37]	Promote grant success for junior biomedical faculty	145 Pathway to Independence Program (PtIP) investigators and 138 K Club investigators	Duke University School of Medicine	Senior faculty mentors and professional grant writing staff provided (1) 20 hours of curriculum via lectures and hands-on workshops, (2) career development counseling, (3) peer groups, and (4) an internal study section
Hackworth *et al.* [25]	Examine the association between mentoring and academic productivity for junior faculty members in a pediatric academic health center	319 Instructors and Assistant Professors for calendar year 2017	Cincinnati Children’s Hospital Medical Center	Assignment of a primary mentor and a Career Development Committee (CDC) designed to define and achieve career objectives, align person career goals with institutional goals, and foster a culture of mentoring through mentorship seminars, mentor training, and support of multiple modalities.
Landsberger *et al.* [38]	Encourage academic productivity in clinical-track faculty	32 Assistant Professors without protected time for research	Department of Psychiatry, Indiana University	Facilitated peer-mentoring group, met weekly, fusing peer-mentoring with traditional junior-senior dyadic mentoring through regular meetings
Mayer *et al.* [39]	Evaluate long term impact among peer group members	33 women faculty at Instructor or Assistant Professor rank	Mayo Clinic in Arizona and Mayo Clinic in Florida	Facilitated peer mentoring: a senior faculty and 3–5 junior faculty met twice each month for 1 year to discuss mutually agreed upon topics
Mumma *et al.* [40]	Examine association between departmental and institutional resources and career development awards	103 responses from vice chairs for research and research directors	Academic emergency departments in USA	Departmental research funding, department resources, and institutional resources – included research funds, secretarial support, research coordinator support, protected time, office space, grant development support
Prendergast *et al.* [26]	Evaluate an enhanced peer mentoring program	22 faculty (4 senior peer advisors and 18 junior faculty)	Academic emergency medicine hospital in Chicago, IL	Facilitated peer group-mentoring platform designed to address self-efficacy, relatedness/inclusion, engagement, and institutional commitment to junior faculty. Senior peer advisors advised groups of 4–5 junior faculty formed around primary area of academic focus (education, research, clinical). Groups created action plans at first meeting and met quarterly. Mentees also gave innovations in scholarship presentations for faculty external to mentoring program.
Rice *et al.* [41]	Report initial results of NHLBI programs to increase diversity - Summer Institute Programs to Increase Diversity (SIPID) and Programs to Increase Diversity among Individuals Engaged in Health-related Research (PRIDE)	52 SIPID participants across 3 cohorts and 152 PRIDE participants across 3 cohorts (204 total)	Participants in SIPID and PRIDE	Programs provide mentoring, didactic, and hands-on experiences to enhance research skills for junior-level faculty underrepresented in the biomedical and behavioral sciences
Shollen *et al.* [42]	Understand behaviors associated with formal and informal mentoring and relationship to satisfaction and productivity	354 faculty respondents to online survey	Full-time, paid medical school faculty at the University of Minnesota	Formal mentors having been assigned to faculty and informal mentors since the time of training and currently
Spence *et al.* [43]	Evaluate outcomes of centralized, cost-sharing design of Independent Investigator Incubator (I3) program	26 diverse junior faculty pursuing research careers in first 3 years of career or in transition from career development award to independent research	Indiana University School of Medicine and CTSI	I3 Mentoring Program included one-on-one mentorship from senior faculty compensated for mentorship time, feedback from committee, mentoring resources, career development workshops, professional grant writer and biostatistical support
Viets *et al.* [44]	Evaluate a culturally centered mentorship model designed for ethnic minority faculty	9 mentees (all URM, 6 women) from various disciplines and 3 senior faculty (1 URM, 2 women)	University of New Mexico School of Medicine’s Institute for Public Health	Biweekly meetings for 3 years to provide technical support and skill development as well as psychosocial support; systematic learning opportunities including seminars; funds to support travel and pilot projects
Voytko *et al.* [45]	Assess the value of a mentoring program specifically for women junior faculty	83 unique mentees and 61 unique mentors	Women Junior Faculty Mentoring Program at Wake Forest School of Medicine	One-on-one mentor–mentee pairs based on desired mentoring topics with additional resources to enhance and support mentoring relationship
Waitzkin *et al.* [46]	Investigate outcomes of mentorship program to train minority junior faculty	29 mentees in first 2 cohorts, 13 new trainees in second cohort	University of New Mexico investigators focused on mental health services research	Minority Research Infrastructure Support Program provides individualized and group learning for junior minority faculty through weekly meetings with experienced faculty members; 5-day institute that occurs annually and training institutes; one-on-one meetings between mentors and mentees matched by a committee; mentee support group conference calls
Welch *et al.* [47]	Conduct a descriptive study of faculty mentoring programs and practices in academic departments of emergency medicine	39 of 135 Department Chairs	Departments of Emergency Medicine in US	Formal mentoring programs, skill-based mentoring, peer mentoring, and mentoring committees. Mentor–mentee pairing primarily based on research interest, career niche or skill assessment, with less consideration on gender or diversity.

*Note*: Based on Medline search of PubMed database conducted on August 3, 2022.

## Results

### Study Selection

The structure of search and search terms for PubMed are shown in Table 1. Based on a search of PubMed, 7,911 articles on mentoring were identified, and 316,513 articles on Academic Medicine were identified (Fig. 1). Combining the two sets of search terms resulted in 1,842 articles for title and abstract review. Following title and abstract screening, 1,464 articles were excluded, and 378 articles moved on to full-text review for eligibility (Fig. 1). Most articles excluded at this phase were due to not being focused on junior faculty or on research. Full-text examination further excluded 350 articles, resulting in 25 articles. An additional hand search revealed two articles, resulting in a total of 27 articles included in this scoping review. Most articles excluded at this phase were due to a lack of measurable outcomes, or outcomes that did not align with this review (e.g., articles addressing satisfaction with mentor). Articles that included only qualitative methods or described the general characteristics of a department without a focus on a specific mentoring program or set of resources to support junior faculty development were also excluded at this point.

### Study Characteristics

Table 2 provides a summary of the 27 articles eligible for inclusion in this review [2,22–47]. All included studies focused on mentoring and supporting research-focused junior faculty in achieving optimal outcomes such as career trajectory, career satisfaction, quality of life, and scholarly productivity namely, publications and grants. Of the 27 included studies, 7 studies focused on junior faculty who were women or from racial/ethnic backgrounds underrepresented in medicine [2,22,30,32,36,39,44].

The studies were heterogeneous in terms of the study goal and objective, study population, study setting, and strategy or approach used for mentoring and supporting junior faculty. Sample sizes ranged from 8 to 1,708. Six studies focused on career trajectory as an outcome [2,22–26]; 5 focused on career satisfaction as an outcome [27–31]; 1 focused on quality of life as an outcome [2]; and 21 focused on scholarly productivity as an outcome [22–26,32–47]. Five studies focused on multiple outcomes [2,23–26]. Three studies focused specifically on women [2,30,39], and four studies focused on junior faculty from racial/ethnic backgrounds underrepresented in medicine [22,32,36,44]. There were no articles that focused specifically on leadership or acquiring leadership positions as an outcome for research-focused junior faculty.

Table 3 highlights the primary findings of each study included in the review relative to the outcomes of interest. Mentoring programs captured promotion through ranks [22–26], with three studies highlighting an increase in number of promotions following the mentoring program [23,24,26]. Mentoring programs also highlighted career satisfaction [2,31], including improved self-efficacy [27], and improvement in satisfaction following participation in mentoring programs [28,30]. Finally, significant academic productivity was highlighted as a result of mentorship programs, including increased number of externally funded grants and publication of peer-reviewed manuscripts [23,24,26,35,38,39,41,43,44,47].

**Table 3.** Summary of findings for articles included in review of mentorship for research-focused junior faculty

Article	Outcome measured				Primary findings
	Career trajectory	Career satisfaction	Quality of life	Academic productivity	
Blood *et al.* [2]		*	*		Most important mentor characteristic identified was availability. Mentorship desired for skills required for advancement (research, lecturing, national recognition, writing and publishing articles, program development/strategic planning).
Boutjdir *et al.* [22]	*			*	41 mentees (35% of those tracked) were promoted to Associate Professor or Professor; 37 (27%) were promoted to Associate Professor; and 4 (3%) were promoted to Professor. A total of 187 peer-reviewed grants were awarded with a success rate of 66% (123 awarded). A total of 1,211 peer-reviewed manuscripts were published.
Bredella *et al.* [23]	*			*	43% of instructors received grant funding; 50% received other awards; 10 instructors were promoted to assistant professor, three of which were underrepresented in medicine
Brown *et al.* [32]				*	Five manuscripts under review or published; 9 extramural grants submitted
Brownson *et al.* [33]				*	199 scholarly manuscripts completed by 55 fellows
Burns *et al.* [34]				*	Former CRTI participants received 262 external grant awards and published 1,035 peer-reviewed manuscripts, 173 book chapters, and 115 review articles.
Chen *et al.* [27]		*			Mentees were satisfied with the mentoring relationship, but many expressed a desire for improved self-efficacy (i.e., better understanding of the criteria for advancement, skills and knowledge for meeting research goals, improved work/life balance, collaboration)
Chou *et al.* [35]				*	A total of 33 had 1–10 published manuscripts (median = 6), an increase in publications by 10.21 (*p* = 0.001). A total of 26 (41%) obtained funding of any kind. All 63 investigators were funded between 2004 and 2016.
Daley *et al.* [36]				*	A total of 18 of 19 URM faculty members completed the program, 15 of the 18 have advanced in their careers and are conducing health disparities research. A total of 7 URM investigators received funds from EXPORT ($429,186) to conduct pilot research, and 5 out of 7 have obtained independent extramural funding (about $4.7 million).
Efstathiou *et al.* [24]	*			*	Mentees were more likely to hold senior faculty positions (*p* = 0.030) and be funded and/or promoted (*p* = 0.030) compared to controls.
Freel *et al.* [37]				*	A total of 61 NIH R grant awards and 38 NIH K grant awards.
Hackworth *et al.* [25]	*			*	37 junior faculty (12% of those tracked) received promotions (5 from Instructor to Assistant Professor; 32 from Assistant to Associate Professor). A total of 233 had at least 1 publication (median = 2); 123 received extramural funding as PI or site PI.
Landsberger *et al.* [38]				*	Prior year academic activities (sum of number of articles published, grants submitted, grants awarded, presentations given) did not differ by whether individuals were in the mentoring group or not; current year totals were four times greater and significantly different (mean 8.67 vs. 1.80, *p* = 0.004)
Mayer *et al.* [39]				*	Peer-reviewed publications increased from a mean of 1.9–3.6 per person 2 years after and 3.8 per person 3–5 years after. All academic products (publications, abstracts, presentations, book chapters) increased from 2 years prior increased from a mean of 7.4–10 per person 2 years after, but were stable at 8 3–5 years after.
Mumma *et al.* [40]				*	Individuals with mentors indicated most inadequate resources were research funds and grant development support, most common barriers were protected time, lack of research peers, and time with mentor. Number of departmental R-level funded researchers was the only variable associated with number of departmental career development awards and number of extramural grant applications.
Nagarur *et al.* [28]		*			Significant improvement in composite satisfaction scores (18 domains each ranked 1–5 with 5 being very satisfied) after completion of program (54.5 vs 65.0, *p* = 0.02). Specific improvement within the following domains: career planning, professional connectedness, self-reflection, research skills, and mentoring skills
Prendergast *et al.* [26]	*			*	In 5 years prior to introduction of program a total of three faculty promotions, compared with seven faculty promoted in 5 years since inception of program. Departmental funding grew from $500,000 with no federal funding to $1,706,479; number of annual peer-reviewed publications remained relatively stable between 2012 and 2017
Rice *et al.* [41]				*	Greater number of mentored-research awards received prior to training and a greater number of independent-investigator awards received after training; total of 130 awards verified in NIH Reporter with 58 obtained after training
Shollen *et al.* [42]				*	No significant relationship between satisfaction with current position and total number of articles or role as PI/co-PI on grants. Those with formal mentors significantly more likely to report role as PI/co-PI (*p* < 0.05). No relationship between formal mentors and articles published and no relationship between informal mentors and total number of articles or role as PI/co-PI
Spence *et al.* [43]				*	Total of 81 publications at 12 months (3.1 publication per mentee), awarded $6.9 million after 1 year and $12.1 million after 2 years (median funding changed from $40,000 to $268,000 per mentee)
Travis *et al.* [29]		*			Having a mentor who was very effective or extremely effective was associated with being very satisfied with career (*p* < 0.001); women and men did not differ in having a mentor or mentor effectiveness
Varkey *et al.* [30]		*			Increase in career satisfaction with academic achievement after program (*p* = 0.001)
Viets *et al.* [44]				*	Increase in productivity between pre-program to post- program: grant applications increased from 3 to 12; publications increased from 11 to 37, and professional presentations increased from 43 to 62.
Voytko *et al.* [45]				*	Consistent promotion, grant applications, grant awards, and manuscripts for mentors and mentees across 5 years of program. No differences by gender of the mentor or length of mentoring relationship.
Waitzkin *et al.* [46]				*	First cohort submitted 31 grant proposals, of which 24 were funded, and published 27 articles. Second cohort submitted 11 grant proposals to date of publication.
Walensky *et al*. [31]		*			Faculty without mentor were more likely to be stalled at rank than those with a mentor who had a low-quality score (*p* < 0.03); those with high mentorship scores had 4-fold odds of reporting strong job satisfaction (*p* < 0.001)
Welch *et al.* [47]				*	Association between higher levels of perceived mentoring success and more NIH funding (*p* = 0.02) and higher publication rates (*p* = 0.02). More NIH funding associated with mentoring relationships in departments that assigned mentors (*p* = 0.02).

### Effective Mentoring Strategies

Table 4 provides a summary of the mentorship format and mentoring program characteristics of each study. The vast majority of programs used mentor–mentee pairs [2,23–25,27–29,31–33,37,41–47], and three programs used primary mentors with committees [25,43,47]. Five programs used a cohort process where mentees moved through the program together [22,33,34,36,41]. Six programs used peer groups [23,25,27,37,46,47], and four programs used facilitated peer mentorship [26,30,38,39]. In addition to regular meetings between mentors and mentees, a number of characteristics and components of programs were highlighted, including didactic courses [22,23,25,33,34,37,41,44,46], career development discussion [22,23,27,29,34,36,37,43], grant development or review of grant applications [22,27,32,35,37,43], manuscript development [30], institutional or national networking opportunities [22,23,26], action plans [24,26,28], and pilot funding or funded support [23,29,32,35,36,40,44,45,47].

**Table 4.** Summary of mentoring program format and characteristics of programs mentoring research-focused junior faculty

Article	Mentorship format					Characteristics of program						
	Cohort	Peer groups	Facilitated peer	Primary mentor with comm.	Mentor-mentee pair	Institute, didactic courses	Career devel discussion	Grant devel or review	Manuscript develop	Institutional or national networking	Action plans	Pilot funding or funded support
Blood *et al.* [2]					x							
Boutjdir *et al*. [22]	x					x	x	x		x		
Bredella *et al.* [23]		x			x	x	x			x		x
Brown *et al.* [32]					x			x				x
Brownson *et al.* [33]	x				x	x						x
Burns *et al.* [34]	x					x	x					
Chen *et al.* [27]		x			x		x	X				
Chou *et al.* [35]					x			X				x
Daley *et al.* [36]	x						x					x
Efstathiou *et al.* [24]					x						x	
Freel *et al.* [37]		x			x	x	x	x				
Hackworth *et al.* [25]		x		x		x						
Landsberger *et al.* [38]			x									
Mayer *et al.* [39]			x									
Mumma *et al.* [40]												x
Nagarur *et al.* [28]					x						x	
Prendergast *et al.* [26]			x							x	x	
Rice *et al.* [41]	x				x	x						
Shollen *et al.* [42]					x							
Spence *et al.* [43]				x	x		x	x				x
Travis *et al.* [29]					x		x					
Varkey *et al.* [30]			x						x			
Viets *et al.* [44]					x	x						x
Voytko *et al.* [45]					x							
Waitzkin *et al.* [46]		x			x	x						
Walensky *et al.* [31]					x							
Welch *et al.* [47]		x		x	x							

Traditional mentoring approaches such as frequent mentor–mentee meetings, attendance at didactic workshops on career-related topics, training sessions on how to optimize mentorship, hands-on experiences to enhance research skills, feedback from committee members, mentee support groups, and individualized assistance with skill development (i.e., grant writing and review; manuscript development) were found effective particularly in terms of academic productivity [23,24,27–31,34,36,41,43,46].

Peer mentoring was used to support professional and personal development skills, relatedness and inclusion, grant writing skills, engagement, promotion, and institutional commitment [23,26,27,30,38,39,45]. Peer mentoring that was matched based on research and clinical skills was found to be effective with regards to increasing satisfaction with academic achievement [30]. When facilitated by a senior mentor, advice was also provided regarding life-work balance, funding opportunities, regulatory issues, and ethical concerns in conducting research [26,34,38]. Individuals participating in peer mentoring were more likely to have improved satisfaction scores, particularly in career planning and research and mentoring skills [28,30].

Using resource support as a mentoring strategy through provision of pilot funding or travel support resulted in increased grant applications, publications, and professional presentations [23,36,40,44]. Junior faculty were also offered additional resources in the forms of research funding, administrative and research coordination support, protected time, office space, and grant development support [40].

Though the majority of programs incorporated formal mentorship, some investigated the impact of informal mentorship [24,42,47]. Formal mentorship resulted in significantly more junior faculty members becoming principal investigators and coinvestigators of research projects (*p* < 0.05) [42]. In addition, higher rates of success for grant funding from the National Institutes of Health among mentees were associated with mentoring relationships in departments that assigned mentors compared to departments where mentors were not assigned (*p* = 0.02) [47]. Informal mentorship, on the other hand, was not associated with increased academic productivity either in grants or manuscripts [42]. In assessments to understand perspectives of mentoring relationships that were successful, junior faculty identified time spent with mentors, varying roles of mentors, and collegiality between the mentor and mentee as important [21].

## Discussion

Though a large literature base exists describing mentorship programs, commenting on the importance of mentorship, and capturing satisfaction with mentorship in general or mentorship programs specifically, this scoping review found there are limited quantitative evaluations of mentorship designed to support diverse groups of research-focused faculty on which to build evidence-based programs. This is an important finding given the large number of published manuscripts identified in the search covering the topic of mentorship in academic medicine. Articles identified in the initial search more often focused on clinical field specific mentorship as opposed to research-specific mentorship (i.e., clinical skills, clinical supervision, procedures, or continuing medical education). In addition, many articles identified in the search discussed mentorship success, but without quantitative evaluation (i.e., editorials, perspectives, commentaries, description of mentoring program without an evaluation, measurement of satisfaction with a program but not evaluation of the program impact). An important recommendation for future research is systematic collection of information to provide evaluations of mentoring program impact. Specifically, information is needed to understand the impact on career trajectory and career progression. Within the articles meeting inclusion criteria for this review, most studies captured research productivity and career satisfaction, with fewer focused on career trajectory and quality of life, and no studies with information collected on progression of mentees to leadership positions.

This scoping review adds to the current literature by offering insights on the state of quantitative evaluations of mentoring programs focused on promoting career success for research-focused junior faculty. Though formal evaluations were limited and difficult to compare, some consistent strategies emerged that are effective for promoting research productivity, career trajectory, and career satisfaction. First, a structured framework for addressing career development gaps was necessary. This framework could include regular meetings, didactic training sessions, or meetings with peers; the type of structure was not associated with outcomes as much as having regular processes in place for mentees. Second, gaps identified spanned research-specific skills such as writing and publishing articles, research-specific resources such as funding for pilot grants and research staff support, as well as career development skills, such as negotiation skills, strategic planning, and balancing work and life. Successful programs should include all aspects to ensure mentees are supported across domains identified as gaps. Third, evaluations were most informative, and programs could address needs most efficiently when they combined both objective measures (i.e. funding and publications) and subjective measures (i.e. networking experiences, confidence in skills, preparedness for research career). Finally, a few specific areas were found successful for ensuring diversity within programs. One, early communication of programs and encouragement of enrollment. Two, incorporating faculty development and leadership development topics within the program. Three, incorporating review and critique of grant applications. And, finally, providing financial support for pilot research projects or travel to national conferences for networking.

Peer mentoring is one strategy that was consistently identified as effective in increasing academic productivity for research-focused junior faculty [26,27,30,38,39]. Two primary types of peer mentorship were noted in the studies included in this review – the first composed entirely of peers (eg., other research-focused junior faculty), and the second best described as facilitated peer mentorship where a senior faculty guides discussions and making himself or herself available to multiple junior faculty mentees. The type of peer mentorship, focus of the program, and number of faculty per peer group varied across studies included in this review, suggesting that the construct of peer mentorship is more important than specific details of the program. Most programs used a facilitated peer mentorship model, which may be based on the need for insight from a more senior faculty member on honing research skills [26,30,38,39]. Based on the findings of this review, programs using peer mentorship should be thoughtful in their design to ensure time expectations for both mentors and mentees are considered. In addition, programs should ensure that group creation and senior mentor matching to a peer group is based on agreed-upon categorization, and goals of the peer mentorship structure are clear. An interesting finding from Shollen and colleagues that informal mentors were correlated with greater junior faculty satisfaction, while formal mentors were correlated with greater academic productivity highlights the importance of formal structures despite the difficulty in matching mentors and mentees [42]. Taken together, these findings suggest that a formal facilitated peer mentorship structure with initial matching into a group based on research interests, followed by regular self-evaluation by mentees allowing for changes in peer mentor groups, may be an effective long-term strategy for effective mentorship. Future research on peer mentorship programs with details on structure developed, processes followed, and career impact on mentees is warranted to guide these programs.

A secondary goal of this review was to identify mentoring strategies that promote diversity by gender and race/ethnicity. Studies in this review found no difference between men and women in terms of having a mentor, mentor effectiveness, or length of the mentor relationship [29,45]. Prior work noted that women faculty reported mentor availability as the most important characteristic for success [2]. They also recommended that the best strategies for their success include mentorship at the relevant career rank and use of structured frameworks to address gaps in establishing and achieving career goals; developing negotiation skills; writing and publishing articles; planning strategically; finding collaborators; and balancing work and family life [2]. In a study for underrepresented minority (URM) junior faculty to create a cohort of investigators engaged in health disparities research, junior faculty reported that early identification of and communication with other URM faculty was a strategy for success [36]. Additional best practice recommendations included enrollment in leadership and faculty development programs and support for pilot research projects (i.e., reviewing, critiquing, funding) [36]. To continue our efforts towards improving workforce diversity among women and/or URM junior faculty members, qualitative and quantitative research dedicated to understanding their needs and identifying best practices and mentoring strategies by gender and race/ethnicity remain an imperative of significant priority. Research collaboratives and partnerships with trusted organizations and minority-serving institutions such as Historically Black Colleges and Universities (HBCUs) should be considered when determining best practices for improving workforce diversity by gender and race/ethnicity for emerging research junior faculty members.

Evidence shows that the emphasis placed on recruiting and retaining women and URM junior faculty in science and health professions has been promising, but unfortunately has shown only modest results [48]. Women and URM junior faculty members are still often overlooked and experience adversities such as structural racism, discrimination, and systemic oppression that inhibit their abilities to get funded, achieve promotion and tenure, and remain in tenure-track positions [48]. This can be compounded when there is limited institutional support, a lack of mentorship, or poor and suboptimal mentoring [48]. Given this lag in the advancement of women and URM junior faculty in research, it is of urgency that we identify the barriers experienced by these subpopulation groups and intentionally initiate strategies and best practices targeted to address the barriers and facilitate their success. In addition, resources and research-intensive training are required to mitigate these barriers and improve the success of women and URM junior faculty in research [48]. Otherwise, evidence suggests faculty diversity will not improve without additional measures and optimal strategies being in place [48].

While many articles aimed to ensure equal representation of men and women in mentorship programs or focus mentoring programs on women junior faculty, fewer programs targeted mentees from racial/ethnic backgrounds that are underrepresented in medicine. This suggests a need to promote recruitment of diverse faculty into ongoing mentorship programs. Mentoring strategies used in programs that targeted diversity can be applied in all programs, such as ensuring that diverse mentors are available, matching mentors and mentees on agreed-upon characteristics and goals, targeting recruitment of minority junior faculty, and combining traditional one-on-one and peer mentoring strategies to create an environment of support. An interesting finding was that while many programs were focused on specific clinical fields, programs that targeted diverse scholars were more likely to engage interdisciplinary mentees with a focus on facilitating research careers across clinical specialties. Current programs should consider increasing the multidisciplinary nature of both mentors and mentees as efforts to increase diversity are implemented.

Based on the current state of research on mentoring strategies to promote career success for diverse scholars, efforts are needed at three levels. First, at the mentee level, programs should focus on purposeful recruitment of diverse mentees and provide mentorship on research competencies, career path guidance, service activity selection, work/life balance, building professional relationships, and acquiring leadership positions. Use of a combined one-on-one and peer mentorship, or a facilitated peer mentorship program will additionally offer a sense of community and shared learning within peer groups, which has been shown to be important for diverse scholars. Second, more research is needed investigating strategies for mentors, including purposeful recruitment of diverse mentors, providing training on expectation setting, conflict resolution, and communication styles to develop the mentor relationship, and use of a mentorship team or external mentors to support the mentor–mentee pair. Finally, strategies are needed at the institutional level to create a sense of belonging and provide access to resources necessary to support research productivity. Resources to support mentors, either offering protected time or dedicated pilot funds to assist mentees, have also been recommended as a way for institutions to show support for mentorship. Efforts to purposely address implicit bias and change systems that limit the long-term success of diverse scholars are necessary not only within the mentor–mentee experience, but also at the departmental and institutional level.

Despite using a systematic process, limitations to this review exist that are worth noting. First, evaluations of mentoring programs not published in the peer-reviewed literature were not captured in this summary. The search purposely used the most widely accepted medical database, Medline, and required peer review to focus on articles that would be most likely to be used as examples for institutions currently developing or revising mentorship programs. Therefore, while other evaluations may exist, those included in this review represent the published literature. Second, most studies noted limitations of small sample size and groups established by self-selection. Additionally, only six studies directly addressed mentoring of diverse junior faculty. While this may impact generalizability of specific programs to other institutions, the summary of characteristics of successful programs should be generalizable given the compilation of multiple programs with similar structures. Finally, this review was purposely focused on mentoring programs conducted within academic medical centers in the USA and therefore may not be generalizable to other settings.

## Conclusion

In conclusion, this scoping review of mentorship programs for research-focused junior faculty highlights the importance of increasing quantitative evaluations of the impact on career success for future design of evidence-based mentoring efforts. Based on studies included in this review, traditional one-on-one mentorship, structured peer mentorship facilitated by a senior mentor, and peer groups in combination with one-on-one mentorship are effective strategies to facilitate research productivity. Few studies focused on programs designed to increase diversity suggesting existing mentorship programs should increase efforts to purposefully recruit diverse mentors and mentees and expand research topics to allow interdisciplinary research engagement. Finally, to achieve long-term impact of these programs, efforts are needed at the mentee, mentor, and institutional level to provide mentorship to junior faculty on research competencies and career trajectory, create a sense of belonging, and connect junior faculty with institutional resources to support career success.
